# Microneedle Array Design Determines the Induction of Protective Memory CD8^+^ T Cell Responses Induced by a Recombinant Live Malaria Vaccine in Mice

**DOI:** 10.1371/journal.pone.0022442

**Published:** 2011-07-25

**Authors:** John B. Carey, Frances E. Pearson, Anto Vrdoljak, Marie G. McGrath, Abina M. Crean, Patrick T. Walsh, Timothy Doody, Conor O'Mahony, Adrian V. S. Hill, Anne C. Moore

**Affiliations:** 1 School of Pharmacy, University College Cork, Cork, Ireland; 2 The Jenner Institute, University of Oxford, Oxford, United Kingdom; 3 National Childrens' Research Centre, Our Lady's Childrens' Hospital Crumlin, Dublin, Ireland; 4 Tyndall National Institute, Lee Maltings, University College Cork, Cork, Ireland; 5 Department of Pharmacology, University College Cork, Cork, Ireland; Universidade Federal de Minas Gerais, Brazil

## Abstract

**Background:**

Vaccine delivery into the skin has received renewed interest due to ease of access to the immune system and microvasculature, however the stratum corneum (SC), must be breached for successful vaccination. This has been achieved by removing the SC by abrasion or scarification or by delivering the vaccine intradermally (ID) with traditional needle-and-syringes or with long microneedle devices. Microneedle patch-based transdermal vaccine studies have predominantly focused on antibody induction by inactivated or subunit vaccines. Here, our principal aim is to determine if the design of a microneedle patch affects the CD8^+^ T cell responses to a malaria antigen induced by a live vaccine.

**Methodology and Findings:**

Recombinant modified vaccinia virus Ankara (MVA) expressing a malaria antigen was percutaneously administered to mice using a range of silicon microneedle patches, termed ImmuPatch, that differed in microneedle height, density, patch area and total pore volume. We demonstrate that microneedle arrays that have small total pore volumes induce a significantly greater proportion of central memory T cells that vigorously expand to secondary immunization. Microneedle-mediated vaccine priming induced significantly greater T cell immunity post-boost and equivalent protection against malaria challenge compared to ID vaccination. Notably, unlike ID administration, ImmuPatch-mediated vaccination did not induce inflammatory responses at the site of immunization or in draining lymph nodes.

**Conclusions/Significance:**

This study demonstrates that the design of microneedle patches significantly influences the magnitude and memory of vaccine-induced CD8^+^ T cell responses and can be optimised for the induction of desired immune responses. Furthermore, ImmuPatch-mediated delivery may be of benefit to reducing unwanted vaccine reactogenicity. In addition to the advantages of low cost and lack of pain, the development of optimised microneedle array designs for the induction of T cell responses by live vaccines aids the development of solutions to current obstacles of immunization programmes.

## Introduction

**Figure 1:**
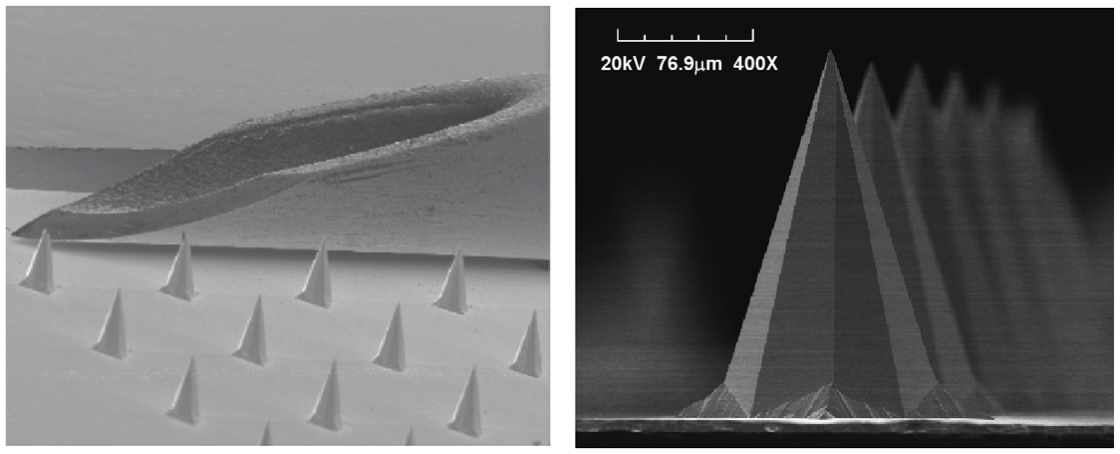
ImmuPatch microneedle arrays. Left: Scanning electron micrograph (SEM) that compares the bevel of a 26G needle to microneedles on an ImmuPatch device. Right: SEM image of individual microneedle.

Vaccination represents the primary public health measure to combat infectious diseases. However estimates of the cost of global immunization programmes to 2015 demonstrate that up to 60% of the total cost of administering some vaccines will be due to systems costs, predominantly cold chain, personnel and training [1]. Use of a simpler vaccine delivery device, which is preferably pain-free, that eliminated sharps waste and reduces the requirements for training in immunization technique and logistics should have a significant positive outcome on the cost and effectiveness of immunization programmes. The intradermal route has demonstrated significant advantages with respect to dose-sparing and immunogenicity in comparison to other routes [2], however ID immunization with needle–and-syringe is technically challenging to administer and inaccurate administration can lead to increased side effects [3], [4]. Skin scarification administration of poxviruses is efficacious, as evidenced by the smallpox eradication campaign and the recent demonstration in mice that delivery of the poxvirus, modified vaccinia virus Ankara (MVA), by skin scarification induced greater protective efficacy against vaccinia virus compared to systemic immunization [5]. However this vaccine delivery method also requires specialised training and equipment and cannot be self-administered [5]. Alternative epidermal abrasion vaccination techniques are being developed, however these can result in increased adverse events compared to intradermal (ID) delivery [6]. Microneedles are micron-scale needles that penetrate the stratum corneum (SC), creating temporary conduits for percutaneous drug or vaccine administration to desired depths in the skin or underlying tissue. Due to their micron sized height, they do not stimulate underlying pain receptors [7]. We developed a method of manufacturing ‘wet-etch’ solid silicon microneedle array patches [8] that produces microneedles with an outer pyramidal profile (**Fig. 1**), termed ImmuPatch. By virtue of their smooth surface and ultrasharp tips, these ImmuPatch devices go cleanly in and out of tissue at very low insertion forces. Thus, ImmuPatch arrays do not abrade the skin, instead they create temporary pores through the stratum corneum (SC) through which vaccine enters the skin.

The microneedle vaccine delivery field has predominantly focussed on antibody induction by subunit vaccines [9]. It was previously concluded, using a subunit vaccine that induction of serum antibody responses was independent of depth of delivery, density of microneedles or area of application [10], thus proposing that the design of the microneedle array has little impact on the induction of immunity. Furthermore, efficacy studies of microneedle-delivered vaccines have centred on mucosal protection, generally of influenza virus respiratory challenge.

Live recombinant viral vectors that induce T cell responses are demonstrating potential as vaccine platforms against diseases such as malaria, tuberculosis HIV and cancer [11]. The investigation of microneedle-delivery of live vaccines has to date been limited to antibody induction by a Japanese Encephalitis virus vaccine and guinea pig CD4^+^ T cell responses to BCG vaccination [12], [13]. Induction of protective, systemic, anti-malarial cellular immunity, particularly mediated by CD8^+^ T cells, has not been investigated in any microneedle live vaccine delivery study, to our knowledge. In this study we examined the use of microneedle arrays to administer recombinant MVA to induce this CD8^+^ T cell response. This replication incompetent poxvirus is demonstrating strong clinical potential in malaria and TB immunization regimes where the intradermal route has been a traditional route of choice [11]. The present study was designed with two main objectives. Firstly, we aimed to determine if microneedle array design impacted on the induction of primary and secondary T cell responses by a live vaccine. The second objective was to determine if microneedle-mediated immunization could increase the immunogenicity and efficacy of homologous, repeated immunization with the same virus vector. Here, we demonstrate that microneedle percutaneous immunization induces increased post-boost CD8^+^ T cell response, compared to intradermal immunization, using a live recombinant vaccine. Secondly, unlike previous studies examining antibody induction, the design of the microneedle array has a significant impact on the magnitude and the memory phenotype of the vaccine-induced CD8^+^ T cell response. Finally, we demonstrate that ImmuPatch delivery of a live vaccine does not induce a pro-inflammatory response, in contrast to ID immunization and therefore may be of particular benefit to reducing vaccine reactogenicity.

## Results

### Microneedle array design influences the magnitude and phenotype of vaccine-induced CD8^+^ T cell response

Using a mouse malaria model of recombinant MVA-PbCSP-induced immunity and protection [14], we determined if the dimensions of microneedle arrays affects the phenotype and magnitude of the vaccine-induced immunity. A range of ImmuPatch microneedle arrays were fabricated that differed in the area of the patch, the density of the microneedles, the height of each microneedle and the total pore volume of each patch. The total pore volume of the microneedle array refers to the maximum volume of the conduits that can be created in the skin after application of a microneedle array and has been arbitrarily defined as small, intermediate or large. Nine array types with increasing total pore volume that differ in microneedle density or height were fabricated (**Table 1**).

**Table 1:** Microneedle Array Designs.

*Microneedle Array*	*Microneedle height*	*Number of Microneedles per Array*	*Array Area (mm^2^)*	*Total volume mm^3^*	*Pore volume range*
A	100 µm	16	29.16	0.0018	Small
F	100 µm	100	29.16	0.0113	Small
B	200 µm	16	29.16	0.0145	Intermediate
D	200 µm	25	29.16	0.0227	Intermediate
E	200 µm	36	29.16	0.0326	Intermediate
C	300 µm	16	29.16	0.0499	Intermediate
G	200 µm	81	54.76	0.0734	Large
T125	125 µm	400	100	0.0902	Large
H	300 µm	36	54.76	0.1123	Large

**Figure 2:**
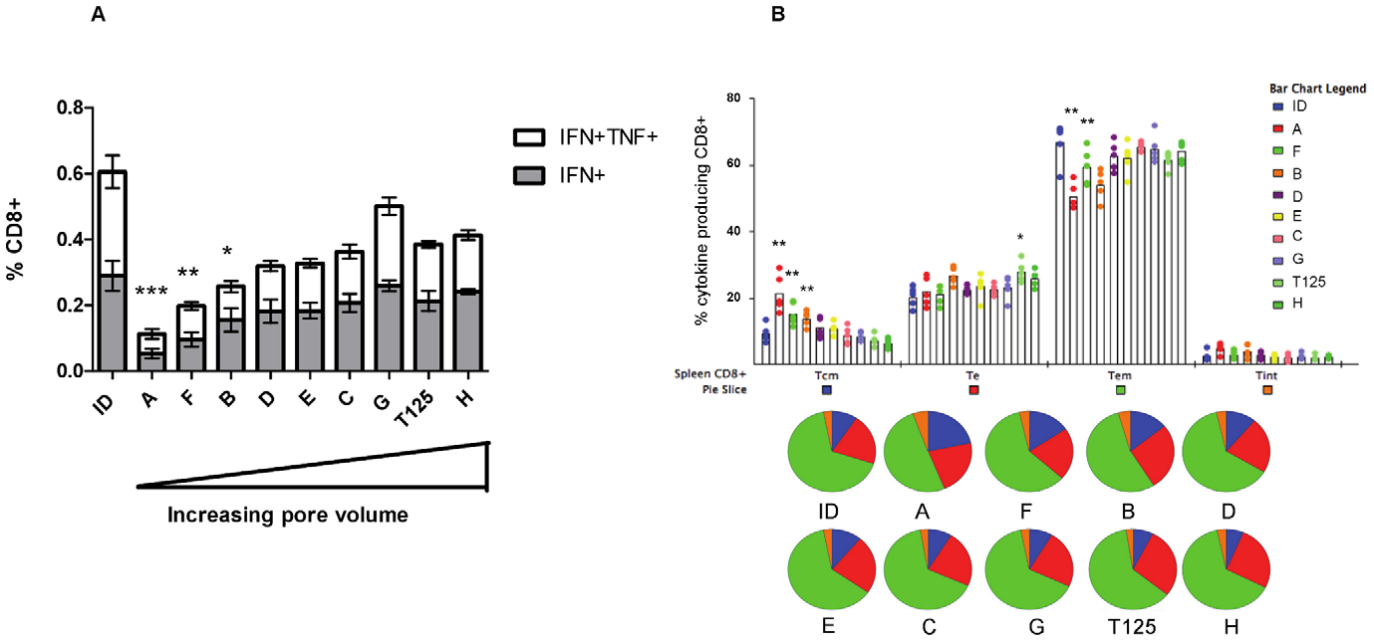
Magnitude and phenotype of the antigen-specific CD8^+^ T cell response after a single immunization. Groups of 5 BALB/c mice were immunized with MVA-PbCSP by the intradermal (id) route or using an ImmuPatch device of increasing pore volume as outlined in Table 1. (**A**) Antigen-specific polyfunctional CD8^+^ T cell responses in spleen cells were quantified after intracellular cytokine staining (ICS) of IFN-γ, TNF-α, and IL-2 subsequent to stimulation with the immunodominant Pb9 peptide. Data are expressed as the frequencies of single cytokine secreting IFN-γ^+^ and dual-secreting IFN-γ^+^TNF-α^+^ CD8^+^ T cells, +/− standard error of the mean (+/−SEM), 2 weeks after immunization. No IL-2 production was observed. (**B**) Memory phenotype of the antigen-specific response. Splenocytes were also stained for CD62L and CD127 in addition to CD8 and ICS of cytokines. The percentage of cytokine-producing CD8^+^ T cells that were central memory T_CM_ (blue pie section), effector T cells, T_E_ (red pie section), effector memory T cells, T_EM,_: (green pie section) and intermediate phenotype, T_int_, (orange pie sections) were determined. Each dot represents one mouse. Graphs were generated after performing a Boolean analysis in FlowJo and data analysis in SPICE software. Pie charts display relative percentages of CD8^+^ T cells that are T_CM_, T_EM_ and T_E_. * p<0.05, ** p<0.01, ***p<0.001 compared with ID vaccinated mice in (A) by one way ANOVA and in (B) by unpaired student t test. Similar results were obtained in four independent experiments.

Female BALB/c mice were immunized with MVA-PbCSP by the intradermal (ID) route or by ImmuPatch-based immunization. The ID route was chosen as it has been repeatedly used in clinical studies and, similar to microneedles, the vaccine is delivered to skin. Two weeks after priming, the frequency, multi-functional quality [15] and memory phenotype [16], [17] of CD8^+^ T cells in the spleen that recognised the dominant, nine-mer, MHC class I epitope in PbCSP, termed Pb9, was determined. Flow cytometry combined with Boolean analysis revealed that two populations of polyfunctional antigen-specific CD8+ T-cell responses were primed by MVA-PbCSP immunization, namely CD8^+^IFN-γ^+^, single cytokine-secreting cells and CD8^+^IFN-γ^+^TNF-α^+^, dual cytokine producing T cells. These populations were approximately equivalent in frequency in each group (**Fig. 2A**, compare IFN^+^TNF^+^ with TNF^+^, **Figure S1**). No other multi-functional phenotype was observed. Of interest, immunization using arrays A and F, that possess the smallest total pore volume, induced significantly decreased frequencies of antigen-specific single and dual cytokine-secreting CD8^+^ T cells, compared to intradermal needle-and-syringe vaccination (ID) (**Fig. 2A**). In contrast, immunization with larger pore volume microneedle arrays induced a T cell response that was equivalent in magnitude and multi-functionality to ID. Vaccination using a flat silicon patch, with no microneedles, resulted in a background T cell response (data not shown), demonstrating that breaching the SC is required to induce immunity.

We used a conventional gating strategy to determine the memory phenotype of antigen-specific CD8^+^ T cells, using the expression of CD62L and CD127 on TAPI-2 treated cells to identify central memory T cells (T_CM_: CD62L^+^ CD127^+^), effector memory T cells (T_EM_: CD62L^−^ CD127^+^), effector T cells (T_E_: CD62L^−^ CD127^−^) and intermediate phenotype T cells (T_int_, CD62L^+^ CD127^−^ ) [16], [17]. Subsequent to a 6 hour stimulation with the Pb9 epitope, examination of the memory phenotype of all antigen-specific, cytokine-secreting T cells demonstrated that, in contrast to ID immunization, the use of a small volume microneedle array induced a significantly higher proportion of central memory (T_CM_) CD8^+^ T cells and significantly reduced effector memory (T_EM_) response (**Fig. 2B** **, Figure S1**). Therefore, immunization with ImmuPatch arrays with small volumes induces an immune response that has a lower frequency of cytokine-producing T cells, which have differentiated to a T_CM_ memory phenotype.

We demonstrate that microneedle height is not the predominant factor responsible for T cell response. Vaccine delivery using T125 arrays, which have microneedles of height 125 µm, similar to patches A and F, but with a pore volume similar to patches G and H, induced a response that was significantly different to A and F but equivalent to patches G and H (**Fig. 2**). This demonstrates that, compared to administration depth, the total volume of a microneedle array is a dominant feature that influences the magnitude and phenotype of the vaccine-induced T cell response. The most significant and strongest positive correlation (Spearman's rho = 0.6859 p<0.0001) was observed between total volume and cytokine producing CD8^+^ T cell responses post-prime. In contrast, other design parameters, such as microneedle height were either not as strongly correlated (Spearman's rho = 0.516, p<0.001) or, in the case of the number of microneedles per array, did not correlate (Spearman's rho = 0.289, not significant) with antigen-specific CD8^+^ T cell responses. Therefore, we demonstrate that total volume of the microneedle array and not microneedle length is a dominant design feature that impacts the type and magnitude of a CD8^+^ T cell response that is induced by a live vaccine.

### Post-boost CD8^+^ T cell responses

**Figure 3:**
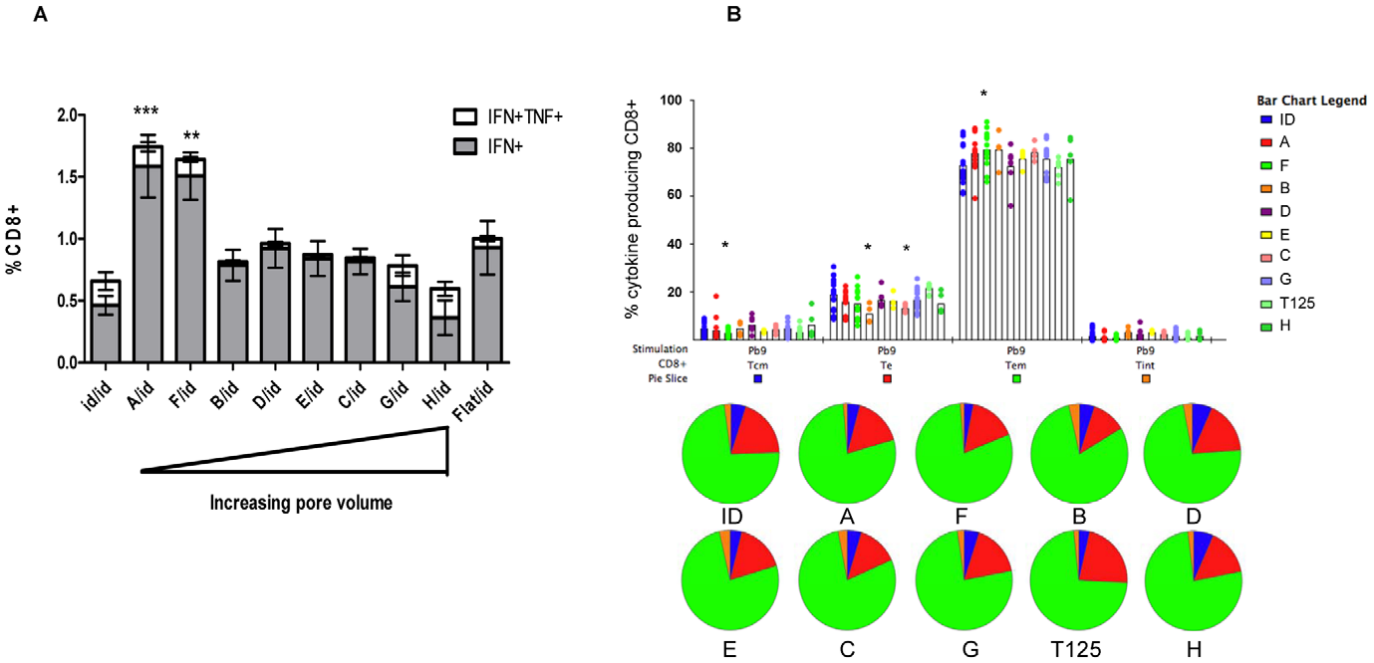
Magnitude and phenotype of the antigen-specific CD8^+^ T cell response after an ID boosting immunization. Groups of 5 BALB/c mice were immunized with MVA-PbCSP by the ID route or using an ImmuPatch device of increasing pore volume as outlined in Table 1. Alternatively vaccine was administered to mice with a silicon patch that was not etched with microneedles, termed ‘Flat’. Two weeks after priming, all mice were immunized with MVA-PbCSP by the intradermal (id) route. (**A**) Antigen-specific polyfunctional CD8^+^ T cell responses in spleen cells were quantified as described in Figure 2, two weeks after the boosting immunization. Data are expressed as the frequencies of single cytokine secreting IFN-γ^+^ and dual-secreting IFN-γ^+^TNF-α^+^ CD8^+^ T cells, +/− standard error of the mean (+/−SEM), 2 weeks after immunization. (**B**) Memory phenotype of the antigen-specific response was determined as described in Fig. 2B. Pie charts display relative percentages of CD8^+^ T cells that are T_CM_, T_EM_ and T_E_. * p<0.05, ** p<0.01, ***p<0.001 compared with ID vaccinated mice in (A) by one way ANOVA and in (B) by unpaired student t test. Data is pooled from four independent experiments (n = 5 mice/group per experiment).

The ability of T cells induced by ID or ImmuPatch immunization to re-expand to a secondary homologous immunization with MVA-PbCSP was examined two weeks after an ID boosting immunization. All groups were boosted in the same manner so that post-boost immunity reflected differences in the existing primary response. Priming using small pore volume microneedle arrays (arrays A and F) resulted in significantly increased post-boost T cell response compared to ID immunization (**Fig. 3A** **, Figure S2**). No significant increase in the magnitude of the T cell response was observed in any other group compared to priming with a flat microneedle patch. The post-boost response was dominated by IFN-γ^+^ CD8^+^ T cells, compared to an equivalent frequency of both cytokine-secreting populations post-prime. A significant negative correlation between pore volume and antigen-specific T cell responses was observed for post-boost total CD8^+^ T cell responses (p<0.0001 r = −0.4786). Thus delivering a vaccine with a small volume microneedle array induces a primary T cell response, with a higher proportion of antigen-specific T cells that are of a T_CM_ memory phenotype that responds vigorously to secondary immunization. Analysis of the total number of antigen-specific CD8^+^ T cells post-prime and post-boost demonstrated a similar pattern as that observed for the percentage of antigen-specific CD8^+^ T cells, for example, priming with the smallest pore volume microneedle arrays induced a significantly decreased number of antigen-specific CD8^+^ T cells post-prime and a significantly increased number of antigen-specific CD8^+^ T cells subsequent to ID boosting (**Figure S3**).

The memory phenotype in all groups subsequent to an ID boost was predominantly T_EM_ (**Fig. 3B** **, Figure S2**). Priming with ImmuPatch array F, but not other small volume arrays, induced a significantly increased proportion of T_EM_ and significantly decreased T_CM_ CD8^+^ T cell phenotype compared to ID immunization post-boost.

### Homologous vaccination using patches in prime and boost

**Figure 4:**
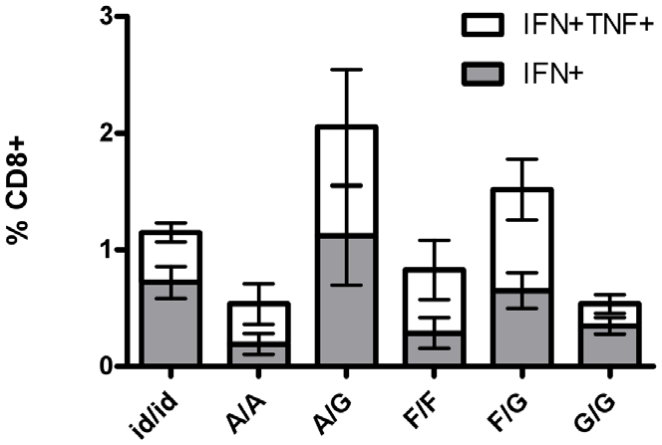
Priming and boosting with ImmuPatch devices induces equivalent or higher T cell responses to homologous ID immunization. Mice were immunized by the ID route or with ImmuPatch arrays A, F or G. Two weeks after boosting, mice were boosted by the ID route (id/id) or boosted using the same array used in the prime (A/A, F/F or G/G) or with array G (A/G, F/G, G/G). Antigen-specific polyfunctional CD8^+^ T cell responses in spleen cells were quantified as described in Figure 2, two weeks after the boosting immunization. Data are expressed as the frequencies of single cytokine secreting IFN-γ^+^ and dual-secreting IFN-γ^+^TNF-α^+^ CD8^+^ T cells, +/− standard error of the mean (+/−SEM, n = 4 mice/group), 2 weeks after immunization. Similar results were obtained in two independent experiments.

In a clinical context, it would be preferable, from a cost and logistic viewpoint, if the same route or delivery device is used in repeated immunizations. To determine if microneedle-mediated vaccination could completely replace needle-and-syringe based delivery, naïve mice were immunized with the leading priming ImmuPatch arrays, A or F, and boosted using the same array or using array G. This microneedle array was chosen as a boosting device as it induced immune responses with similar memory and functional phenotype to needle-and-syringe post-prime (**Fig. 2**). The magnitude and multi-functionality of the T cell response induced by a patch/patch prime-boost regime was equivalent to that induced by repeated ID immunizations (**Fig. 4**). An immune response that was primed using array A or F and boosted with microneedle array G resulted in increased T cell responses, however this was not significantly greater than the ID/ID group. Therefore, the repeated use of a needle-and-syringe can be eliminated by microneedle arrays that can induce equivalent or higher CD8^+^ T cell responses to vaccine antigen post-boost.

### Protective efficacy against a malaria sporozoite challenge

To assess whether the vaccination method impacted on the protective efficacy of a homologous MVA-PbCSP immunization regime, immunized mice were challenged with *P. berghei* sporozoites two weeks after the second immunization (**Table 2**). The level of sterile protection and the time to 0.5% parasitemia were determined. Two separate challenge studies were pooled in this analysis (n = 5 mice/group per challenge study). We first determined if the immunization regimes responded in a similar manner in repeated challenge studies. No significant differences were observed between the two challenge studies for any group except group H/id, thus this group was removed from evaluation of protective efficacy. Significant differences in the hazard ratio compared to that of the naïve group, were determined (far right column). This demonstrated that immunization with A/ID, F/ID, F/G and ID/ID induced a significantly greater reduction in the risk of becoming parasitaemic, compared to unimmunized control mice. In contrast, other immunization groups had a similar risk as the naive mice of developing blood stage parasitaemia following sporozoite infection. There are no significant differences between ID/ID and the protective ImmuPatch regimes (A/ID, F/ID and F/G), however using ImmuPatch-mediated priming and boosting (F/G) resulted in the most reduced risk of becoming parasitemic (hazard ratio = 0.18). Overall, these challenge studies demonstrate that ImmuPatch priming or ImmuPatch prime/boosting is as efficacious as needle-and-syringe delivery of repeated MVA-PbCSP immunizations, resulting in protection against infection or a delay in the progression to blood-stage malaria i.e., partial protection.

**Table 2:** Microneedle-mediated immunization induces efficacy against *P. berghei* sporozoite infection.

Immunization Regime (Prime/Boost)	No. protected/total	Protection (%)	Median time to 0.5% parasiteamia in days	Hazard Ratio (95% CI)^a^	p-value^b^
Naïve	0/10	0	6.52	-	-
ID/ID	4/10	40	6.91	0.25 (0.08–0.81)	0.020*
A/ID	3/10	30	6.76	0.33 (0.11–1.00)	0.050*
F/ID	5/10	50	7.46	0.25 (0.08–0.80)	0.020*
F/G	5/10	50	6.88	0.18 (0.05–0.68)	0.011*
B/ID	4/10	40	6.49	0.39 (0.13–1.16)	0.090
D/ID	2/10	20	6.61	0.44 (0.16–1.22)	0.120
E/ID	3/10	30	6.38	0.59 (0.22–1.59)	0.300
C/ID	4/10	40	6.55	0.61 (0.22–1.74)	0.360
G/ID	4/10	40	6.47	0.39 (0.13–1.17)	0.090
H/ID	6/10	60	nd^c^	0.04 (0.005–0.32)^c^	0.002^c^

Mice were immunized and challenged and protection results analysed as described in Methods.

a Compared to Naïve, unimmunized mice (95% confidence interval);

b compared to Naïve,

*p≤0.05.

c Not determined or unable to determine significance compared to naive group as repeated challenge studies were significantly different.

### Microneedle mediated immunization does not induce pro-inflammatory innate responses

**Figure 5:**
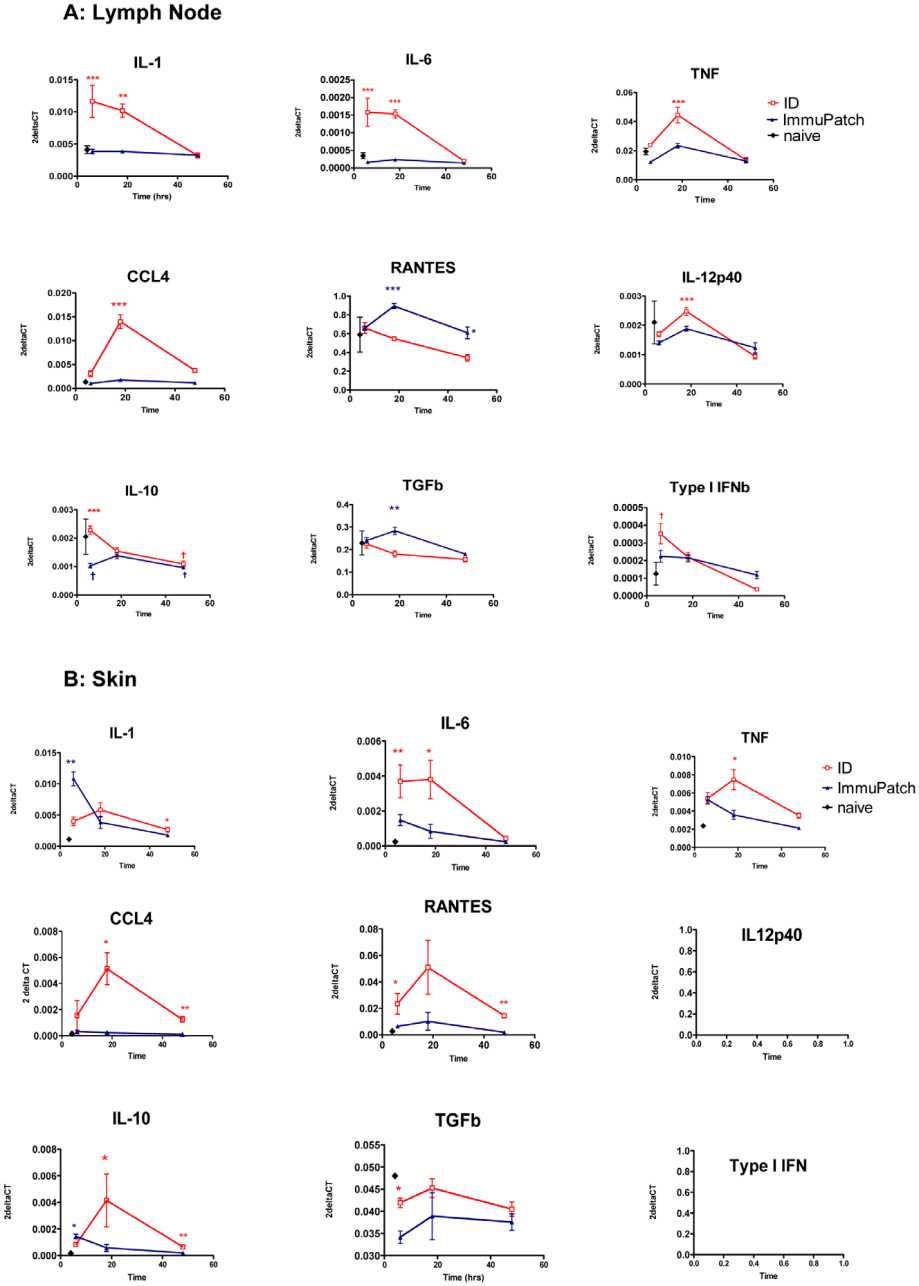
Induction of cytokine and chemokine mRNA in the skin and draining lymph node by ID or ImmuPatch vaccine delivery. Mice were immunized with MVA-PbCSP by the ID route or using ImmuPatch array F at time 0. At 6, 18 and 48 hours after immunization, 5 mice per vaccinated group were culled and the skin site of immunization and the draining lymph nodes were harvested and snap frozen. Skin and lymph nodes from 2 naïve mice were harvested to determine background levels of expression. Gene expression values relative to GAPDH were calculated as 2^−ΔCt^. The mean (+/− SEM) values for each group are plotted; cytokine and chemokine induction due to ID immunization is in red; due to ImmuPatch immunization is in blue and naives is in black. * p<0.05, ** p<0.01, ***p<0.001 by unpaired student t test of the two immunized groups, ^†^p<0.05 compared to naïve mice. Similar results were obtained in two independent experiments.

We wished to determine some underlying mechanisms responsible for the microneedle-mediated modulation of antigen-specific CD8^+^ T cell immunity. In contrast to other transcutaneous immunization studies, ImmuPatch delivery did not rely on skin pre-treatment, such as shaving, as we delivered vaccine to the part of the ear that lacks fur, or on the use of high velocity applicators that stress the skin. We hypothesised that ImmuPatch-mediated vaccination did not induce a pro-inflammatory response post-immunization, thereby permitting the generation of a strong T_CM_ response [18], [19]. We examined the induction of cytokine and chemokine mRNA in the lymph nodes and at the site of immunization, up to 48 hours post-immunization with MVA-PbCSP, as a marker of local inflammatory responses to immunization (**Fig. 5**). Pro-inflammatory cytokines such as IL-1 and IL-6 were induced to a significantly higher level, compared to ImmuPatch-treated mice, in lymph nodes of ID immunized mice at 6 and 18 hrs post immunization. A second wave of gene transcription of pro-inflammatory cytokines and chemokines, specifically TNF-α, CCL4 and IL-12p40, occurred at 18 hrs post-ID immunization that, for the first two molecules were significantly greater than lymph nodes from ImmuPatch-treated or naive mice. Apart from RANTES, no significant changes in pro-inflammatory cytokine induction were observed when vaccine was delivered with ImmuPatch compared to lymph nodes of naïve mice (**Fig. 5A**). A similar pattern of early and strong pro-inflammatory mRNA induction in the skin was observed for most cytokines in ID immunized compared to patch-immunized mice (**Fig. 5B**). However, ImmuPatch mediated immunization resulted in significantly higher IL-1 and IL-10 and equivalent TNF-α mRNA in the skin compared to ID immunization at 6 hours. No mRNA transcript for IL-12p40 or type I IFN could be detected at the site of immunization. Furthermore, higher levels of TNF-α protein was detected by standard cytokine ELISA in the serum at 18 hrs after ID (5.8+/−3.3 pg/ml) compared to ImmuPatch immunization (1.4+/−1.3 pg/ml, mean +/− SEM, n = 4 mice per group). Analysis of other cytokine proteins, such as IL-1 and IL-6, in serum was at the limit of detection of ELISA. These data demonstrate that microneedle-mediated immunization did not induce a classical pro-inflammatory response that was observed subsequent to ID immunization.

**Figure 6:**
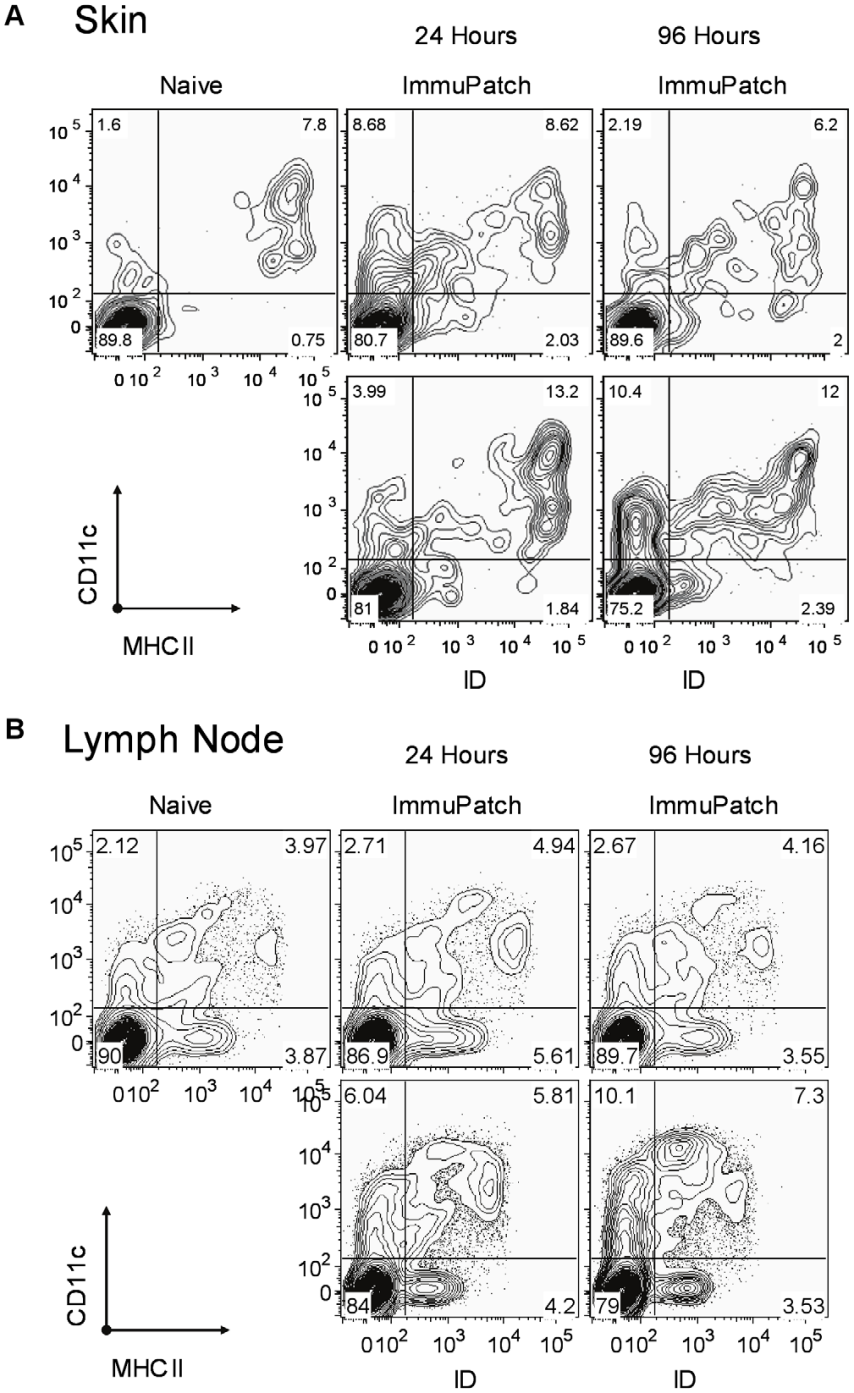
Cell trafficking at the site of immunization and lymph node subsequent to ImmuPatch or ID vaccination. Cells were isolated from the site of immunization (A), or lymph node (B), from naïve mice (upper left panel) or mice immunized with MVA-PbCSP using ImmuPatch array F (upper middle and upper right panels) or ID (lower middle and lower right panels) at 24 (middle panels) or 96 hours (right panels) post-administration. The expression of CD11c and MHC class II was assessed on live CD3- and CD19- cells. Each plot is representative of 3 samples per group. Similar results were obtained in two independent experiments.

We next examined if the observed differences in cytokine and chemokine induction at the site of immunization and lymph node impacted on trafficking and activation of the CD11c+ or CD11c- subset of antigen presenting cells (APC). The total number of cells in the lymph node was consistently three-fold higher in ID compared to ImmuPatch treated and naïve mice (**Figure S4**). Modest increases were observed in the numbers of CD11c+ cells and MHC class II expression in the lymph node after ImmuPatch immunization compared to naïve mice at 24 hours (**Fig. 6**). In contrast, ID administration of MVA caused a more substantial increase in CD11c+MHCII+ and CD11c+MHCII- cells in the lymph node at both 24 and 96 hours post-immunization. A similar effect was noted at the site of immunization; ImmuPatch delivery of MVA resulted in a transient increase in CD11c+MHCII- cells at 24 hrs and CD11c-MHCII+ cells at 24 and 96 hrs, while the proportion of CD11c+MHCII+ cells was similar to naïve at both times. In contrast, ID delivery induced a substantial increase in CD11c+MHCII+ cells that were maintained in the ear up to 96 hrs post-vaccination. These changes in APC numbers and MHCII expression are consistent with increased inflammatory chemokine and cytokine expression (**Fig. 5**).

### Biodistribution due to ID or ImmuPatch delivery

Next we wished to determine if the administration route impacted on the biodistribution of the delivered material. Firstly, we histologically determined that microneedle-delivered material is distributed mainly in the epidermis, with some delivery to the dermis, 30 minutes after administration of red fluorescent beads (100nm diameter) to mice ears using Array F (**Figure S5**). A similar distribution was observed when MVA expressing red fluorescent protein (MVA-RFP) was administered to ex vivo porcine skin. In comparison to ID injection of MVA-RFP, where transfected cells were observed in a dermal bolus, a tract of mostly epidermal infected cells was observed subsequent to ImmuPatch Array G administration (**Figure S6**).

Secondly, we determined the amount of red fluorescent beads that can be recovered from inside the skin of mouse ears subsequent to ImmuPatch administration. To lessen the effects of biodistribution, we harvested ears 30 minutes after treatment. Approximately 3% of the original material was delivered into the ear (**Figure S7**). No significant difference in the percentage recovered was observed between microneedle designs in this study, at this early timepoint.

**Figure 7:**
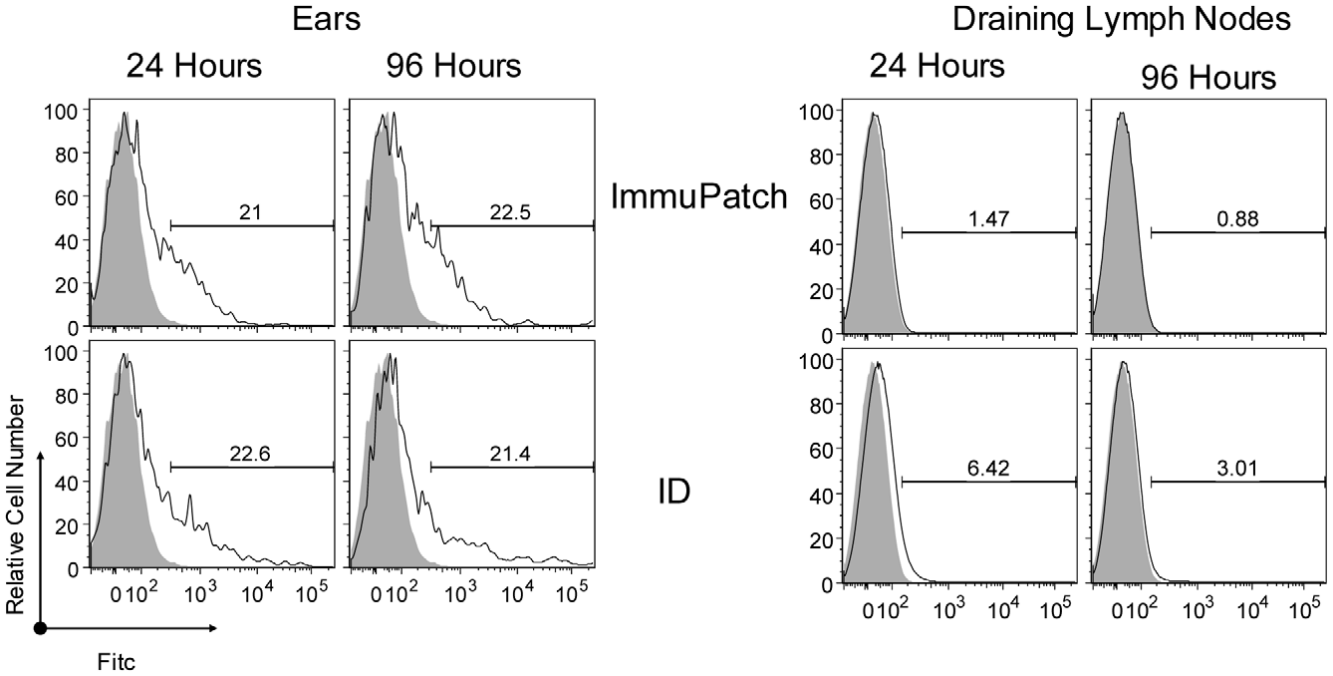
Biodistribution and clearance subsequent to ID or ImmuPatch delivery. FITC (1mg/ml) was added to MVA-PbCSP and administered to mice by the ID route or using ImmuPatch array F. At 24 and 96 hours ears and lymph nodes were harvested from 3 mice per group. The level of FITC detected in live cells from the skin and lymph nodes of ImmuPatch (top panels) or ID (lower panels) treated animals at 24 and 96 hours post-immunization. Vaccinated mice are represented by the bold line, naive mice by the shaded histogram. The plots are representative of 3 mice per group and of 3 repeated experiments.

Finally, in the absence of having an MVA that highly expressed a fluorescent marker that could be easily detected by flow cytometry, we administered FITC mixed with the vaccine. A similar frequency of live cells in the ear contained FITC at 24 and 96 hours in both groups (**Fig. 7**). However, ID delivery resulted in higher levels of FITC in the lymph node compared to ImmuPatch. Therefore, both ID and ImmuPatch administration result in sustained presence of the delivered cargo up to 96 hrs in cells at the site of administration, however ID delivery resulted in greater transport to the lymph node.

**Figure 8:**
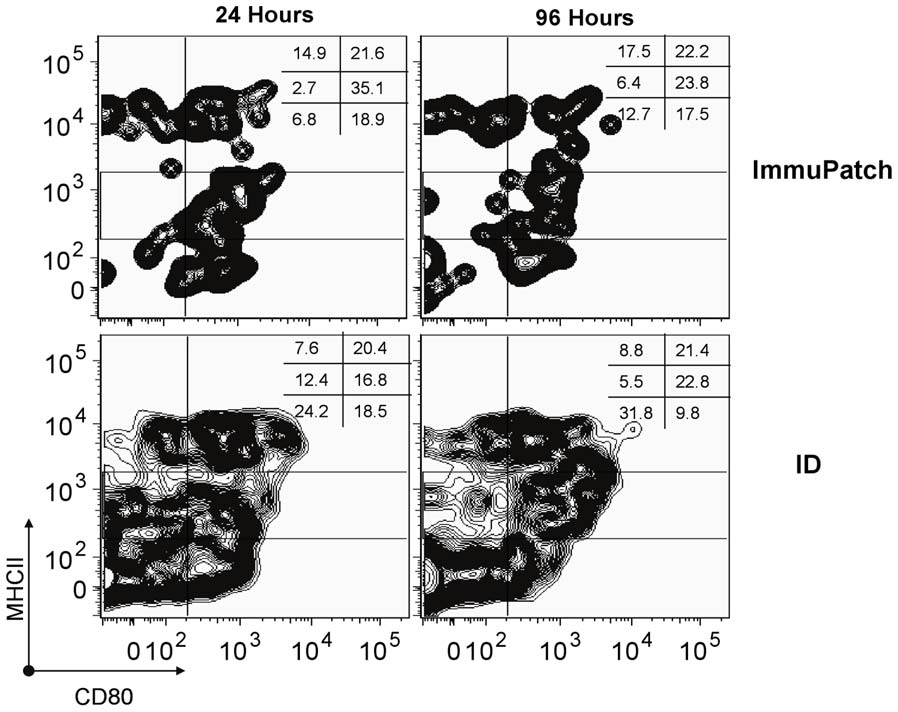
Antigen presentation capability subsequent to ID or ImmuPatch delivery. FITC and MVA-PbCSP was administered to mice and monitored as described in Figure 7. The expression of MHC class II and CD80 on FITC^+^ CD3^−^CD19^−^ live cells was determined in the lymph node of treated animals. Numbers in the upper right hand of each plot represent the percentage of live CD3^−^CD19^−^ cells that express high or positive levels or are negative for CD80 and MHC class II. The plots are representative of 3 mice per group and of 3 repeated experiments.

We next examined the frequency of cells that had taken up FITC that were capable of delivering signal 1 (MHC class II) and signal 2 (CD80) to T cells in the lymph node (**Fig. 8**). We gated out CD3^+^ T cells and CD19^+^ B cell and focused on live FITC+ cells that were MHCII^+^ and/or CD80^+^ that can be viewed as capable of antigen presentation and co-stimulation. Similar frequencies of FITC+MHCII^hi^CD80^hi^ cells were detected in both groups at both times. ImmuPatch treated mice had twice the frequency of MHCII^+^CD80^+^ cells compared to ID at 24 hours (35% compared to 16.8%). A higher proportion of FITC+ cells that were negative for both CD80 and MHC class II were detected in ID treated compared to ImmuPatch treated animals (24% compared to 6% at 24 hours).

Therefore, although the proportion of cells that are FITC+ in the lymph node is lower in ImmuPatch versus ID-treated mice, the frequency of FITC+ cells capable of delivering signal 1 and 2 from the same cell is equivalent in both groups and ImmuPatch treated mice have fewer cells incapable of delivering either signal 1 or 2 to T cells.

## Discussion

Development of low cost, needle-free, painless, safe, efficacious immunization strategies is an important goal in global health care. ImmuPatch is a microneedle-based technology that is being developed as an easy-to-use, pain-free, patch that, by creating temporary channels through the impermeable stratum corneum, delivers vaccine through the skin. Here we aimed to determine if the patch design impacted on the magnitude and phenotype of the vaccine-induced CD8^+^ T cell response. We demonstrate that administration of a live recombinant vaccine using these wet-etched silicon microneedles induces equivalent or significantly greater CD8^+^ T cell responses compared to needle-and-syringe intradermal delivery. In contrast to previous studies that examined microneedle array design in the context of antibody induction [10], we demonstrate that microneedle design significantly impacts on the magnitude and memory phenotype of the vaccine-induced CD8^+^ T cell response and subsequent protection against liver-stage malaria challenge. We also demonstrate that in contrast to needle-and-syringe delivery, microneedle-mediated vaccination does not induce classical inflammatory responses, suggesting that ImmuPatch vaccine delivery could potentially eliminate unwanted reactogenicity.

Here we demonstrate that ImmuPatch vaccine delivery eliminates most of the skin inflammatory response and significantly reduces inflammatory markers in the draining lymph node that is observed by ID vaccination. We propose that this is likely due to the lack of abrasion, irritation and stress of the skin, features that are common to previous epidermal drug or vaccine targeting, such as skin scarification [5], or stripping [6], [20] that tear the SC from the skin and expose the underlying epidermis, or alternatively, biolistic targeting using gene guns or spring-loaded applicators. In contrast to these disruptive methods that can result in systemic adverse events [6], microneedle administration creates temporary channels through the SC [7], [21], [22]. Furthermore ImmuPatch delivery does not cause shear stress during application as no high velocity applicators are required for the microneedles to penetrate the SC. We propose that increased IL-1 and equivalent TNF-α mRNA expression in the skin of ImmuPatch vaccinated mice could be due to MVA infection of keratinocytes that constitute the majority of the epidermis and are major producers of these cytokines [2], [23]. The delivery method also impacted on biodistribution. Injection of a bolus fluid into the dermis causes increased local pressure and capillary permeability and permits efficient antigen drainage into lymphatic capillaries that form an extensive network in the dermis [2], [24], [25], [26]. In contrast, ImmuPatch delivery resulted in equally efficient uptake of the marker in the skin, however the fluorescent label did not drain as effectively to lymph. Also, in contrast to ID delivery, ImmuPatch-administered cargo is delivered equally to the epidermis and to the dermis (**Figure S5, Figure S6**). We suggest that microneedle array targeting to individually created pores in the epidermis and upper dermis does not result in increased interstitial pressure and subsequent lymphatic drainage. However, despite decreased lymph node drainage subsequent to ImmuPatch delivery, equivalent frequencies of FITC^+^ cells capable of delivering stimulatory TCR and co-stimulatory signals to naïve T cells were observed in draining lymph nodes in both immunized groups. Overall this demonstrates that antigen presentation occurs in ImmuPatch-immunized animals in the absence of strong inflammatory responses.

We propose that the increased proportion of T_CM_ is due to the lack of inflammation post-ImmuPatch immunization. It has been previously demonstrated that curtailment of inflammation during the initiation of immunity results in accelerated T cell memory generation [27] and that the default differentiation memory generation pathway for CD8^+^ T cells is deflected by sustained inflammation [18]. Secondly, ID immunization resulted in a marked, sustained increase in level of CD11c^+^MHCII^+^ cells at the site of immunization, whereas ImmuPatch immunization did not. We propose that this continued presence of activated APC, that are capable of sending signals 1 and 2 to T cells in the skin may influence the induction of more T_EM_ and T_E_ cells as antigen persistence is known to maintain CD8^+^ T cells in an effector state [28], [29].

We observed a negative correlation between pore volume and T_CM_ suggesting that antigen load can be optimised for the induction of memory CD8^+^ T cell responses. Of note, total pore volume and not microneedle length was the dominant feature that influenced the magnitude and phenotype of the induced T cell response. This was particularly evident when arrays A and F are compared to array T125. Total array volume is determined by height (as a variable in the equation of a circular cone) and number of microneedles per array and therefore permits the determination of these combined parameters on CD8^+^ T cell vaccine-induced immunity. We propose that the total microneedle array volume is the key design feature that should be examined for specific T cell responses post-vaccination. We propose that total pore volume most accurately reflects the total amount of vaccine that is available to the immune system. We speculate that a lower live virus vaccine dose, delivered by small pore volume patches, favours a T_CM_ response, however as the availability of antigen increases with increasing total pore volume, the response begins to more resemble a bolus administration; as is observed in Figure 2. Further studies are required to determine how such subtle differences in microneedle design are involved in the observed differences in memory induction by different ImmuPatch devices.

Previous efficacy studies involving microneedle-delivered vaccines have focussed on mucosal protection, predominantly assessing influenza virus respiratory challenge [9], [30]. In contrast, here we are assessing systemic cell mediated immunity against liver-stage malaria as the protective mechanism [31]. Defining the optimal microneedle design that promotes systemic cell-mediated efficacy is a novel area of investigation that has future clinical consequences. Homologous MVA-PbCSP immunization induces weak efficacy and as such, represents a vaccine model that can be used to design methods that improve both immunogenicity and efficacy [14]. Despite significantly greater induction of CD8^+^ T cell responses in the spleen by small pore volume ImmuPatch microneedle arrays, no significant differences in efficacy were observed between ID and ImmuPatch immunization when mice were challenged 2 weeks after boosting. This may reflect differences in immunity in the spleen compared to the liver or the phenotype of a protective immune response. It highlights our incomplete understanding of correlates of protective immunity against liver stage *Plasmodium* infection and the necessity of looking at efficacy in addition to immunogenicity. Overall, the result demonstrates that a small pore volume ImmuPatch device, that induces a higher proportion of CD8^+^ T cells with a T_CM_ responses post-prime (compared to ID), should be chosen to maximise efficacy against liver-stage malaria.

Therefore, ImmuPatch mediated immunization is a viable alternative to needle-and-syringe based administration of a T cell inducing live vaccine that reduces innate inflammation, with potentially reduced reactogenicity. The design of the microneedle device impacts on the magnitude and phenotype of the induced immunity and efficacy and should be optimised for use with a CD8^+^ T cell inducing live vaccine. This finding underlies future studies to develop coated or dissolvable microneedle systems incorporating vaccines that induce cell-mediated immunity. In human studies to date (data not shown and refs [7], [22]) no adverse events or pain have been reported using these microneedle arrays. In combination with other advantages of this system, including the lack of pain, the elimination of sharps waste and the capacity to affordably mass manufacture these microneedles, ImmuPatch mediated vaccination demonstrates potential as a feasible needle-free approach to vaccination that aims to overcome several cost and logistic obstacles of immunization programmes.

## Materials and Methods

### ImmuPatch microneedle patch design

Silicon microneedles were fabricated using wet-etch technology as previously described [8]. The area of each microneedle patch and the length and number of microneedles per patch were designed to produce a microneedle patch that created specific total pore volumes when inserted into skin. The pore volume of these pyramidal silicon microneedles was determined using the formula to calculate the volume of a right circular cone. The total pore volume per array is the sum of the volume of each pore on the array. The specific dimensions of each patch is detailed in **Table 1**.

### Vaccines

The construction, design and preparation of Modified Vaccinia Virus Ankara (MVA) expressing *P. berghei* CSP (MVA-PbCSP) and red fluorescent protein (MVA-RFP)) have been previously described [32]. All viruses were resuspended in endotoxin-free PBS for immunization.

### Animals and Immunization

Female BALB/c mice 4–6 weeks old (Harlan UK) were used in all experiments which were conducted in strict accordance with the terms of licences from the Irish Department of Health and Children, under the Cruelty to Animals Act (licence numbers B100/4034 and B100/3157) and the UK Home Office, under the terms of the Animals (Scientific Procedures) Act 1986 (licence numbers 30/7793 and 30/2414)and according to the approval of the UCC AECC and University of Oxford Animal Ethics Commitees. Mice were immunized with 1×10^6^pfu MVA-PbCSP in phosphate buffered saline (PBS) (Sigma). Vaccine was administered with a conventional 28G needle and syringe intradermally (ID) into the ear (50 µl of 1×10^6^pfu per mouse). Alternatively, 5 µl of vaccine was placed on the dorsal surface of the ear and administered to the anaesthetised mouse by pressing a microneedle array onto the ear, using a force of approximately 10–20N (1×10^6^pfu in 10 µl per mouse). Mice were primed on day 0. Post-prime T cell responses were analysed in the spleen on day 14 after immunization. Mice were boosted by the ID route or using a microneedle array at day 14 post-immunization. Vaccine-induced immunity was tested in the spleen of all groups 14 days after boosting.

### Immunogenicity Studies

T cell responses to the dominant MHC class I epitope Pb9 (SYIPSAEKI) [14] were analysed by intracellular cytokine staining and flow cytometry (ICS) in the same method as previously described [17], [33]. Briefly, ACK-treated splenocytes were pre-incubated for 1 hour with TAPI-2 (4 µg/well, equivalent to 100 µM peptide, Peptides International [34] and subsequently incubated for 5 h in the presence of 1 µg/mL Pb9, 100 µM TAPI-2 and 2 µL/mL Golgi-Plug (BD Biosciences). Staining antibodies were specific for mouse and purchased from eBioscience. After blocking Fc receptors with anti-CD16/CD32, cells were surface stained for 30 min at 4°C with Pacific Blue-labeled anti-CD8α and APC-Alexa Fluor 700-labeled anti-CD4, PE-Cy7-labeled CD127 and PerCpCy5.5-labeled CD62L. Cells were permeabilized in Cytofix/Cytoperm solution as per manufacturer's instructions (BD Biosciences). Intracellular cytokines were stained with APC-labeled anti-IFN-γ, FITC-labeled anti-TNF-α, and PE-labeled anti-IL-2. Flow cytometric analyses were performed using an LSRII (BD Biosciences) and data were analyzed with FlowJo (Tree Star) software. One million events per sample were acquired. Analysis of multifunctional T cell responses was performed by using Boolean analysis in FlowJo software and SPICE 4.0 (M. Roederer NIH, Bethesda). Three major subsets of cytokine- expressing, antigen-specific T cells were defined according to their expression of CD62L and CD127 [16], [17]. These markers are associated with central memory T cells (T_CM_: CD62L^+^ CD127^+^), effector memory T cells (T_EM_: CD62L^−^ CD127^+^), effector T cells (T_E_: CD62L^−^ CD127^−^) and intermediate phenotype T cells (T_int_, CD62L^+^ CD127^−^ ).

### Sporozoite challenge and survival analysis

Mice were challenged intravenously with 1000 *Plasmodium berghei* sporozoites (ANKA 234) as described previously [14], [17]. Giemsa-stained blood smears were screened to day 20 post-challenge and % parasitaemia ascertained [35]. Linear regression analysis was used to determine the timepoint at which parasiteamia would reach 0.5% in parasitic mice. A Cox's Proportional Hazards Regression model was used to test for significant differences in the chance of reaching the 0.5% parasitaemia threshold at any one timepoint (assuming that this ratio is the same at each time point) as compared to the naïve control group during the monitoring period.

### FITC uptake and cell trafficking

FITC (1mg/ml solution in PBS) was added to MVA-PbCSP and administered to mice by the ID route or using ImmuPatch array F in the same manner as used for immunization. At 24 and 96 hours after delivery ears and lymph nodes were harvested from 3 mice per group per timepoint. Cells were isolated from ears by modifying a previously described method [36]. Briefly ears were separated into dorsal and ventral leaflets, placed dermal side down in supplemented RPMI1640 media, scored with a blade and scraped to encourage release of cells from the tissue. Following one hour incubation at 37°C, 5% CO_2_, cells were collected and passed through a 70 µm strainer, centrifuged and re-suspended in PBS. Similarly, the LNs were homogenised through a 70 µm cell strainer, centrifuged and re-suspended in PBS. Sample cell suspensions were blocked with Fc-block (BD) and subsequently stained with anti-mouse CD3 and CD19 to gate T and B cells, CD11c, MHC class II (IA/IE) and CD80 (ebioscience). A live/dead cell stain (Invitrogen) was included. Cells within the live cell gate were then analysed for levels of FITC or surface markers.

### RT-PCR assessment of cytokine and chemokine induction

Ears and lymph nodes were snap-frozen in liquid nitrogen immediately after harvesting. Tissues were then disrupted using MagNA Lyser Green Beads and total RNA purified using High Pure RNA tissue kit following the manufacturer's protocol (Roche, Germany). cDNA was prepared from isolated RNA using The High Capacity cDNA Reverse Transcription Kit (Applied Biosystems, USA). Real-time quantitative RT-PCR analysis of cDNA samples, prepared from isolated RNA, for selected genes were performed using the TaqMan® Gene Expression Assays and ABI7300 Real time PCR System instrument and software (Applied Biosystems) following the manufacturer's protocols. The relative expression of the following genes was measured: TNF-α (ID: Mm00443258_m1), IL1-α (ID: Mm00439620_m1), IL-1β (ID: Mm00434228_m1), IL-6 (ID: Mm00446190_m1), IL-10 (ID: Mm00439616_m1), TGF-β1 (ID: Mm00441726_m1), Ccl4 (ID: Mm00443112_m1), Ccl5 (ID: Mm01302428_m1), IL-12β (ID: Mm00434174_m1), IFN-β (ID: Mm00439552_s1). The housekeeping gene used was GAPDH (ID: 4352932E). Real-time PCR data were analyzed as follows: cycle numbers at threshold crossing (Ct) values were subtracted from Ct values for a control housekeeping gene, GAPDH, to generate ΔCt values. Gene expression values relative to GAPDH were calculated as 2^−ΔCt^.

### Statistical Analysis

Data were analyzed using GraphPad Prism version 5 for Windows (GraphPad Software, San Diego, California, USA). Normality of was assessed by Kolmogorov-Smirnov test. Unpaired two-tailed Student's t-test or one way ANOVA were performed, as appropriate, to compare the responses between groups. Cox's Proportional Hazards Regression analysis was preformed using STATA.

## Supporting Information

Figure S1 **Sample FACS plots** **Figure 2** **.** Mice were immunized by the ID route (top panels) or using microneedle Array F (middle panels) or Array G (lower panels). (**A**) Magnitude of the multi-functional TNF-α (Y-axis), IFN-γ (X-axis) response in gated live CD8^+^ T cells that were unstimulated (left panels) or stimulated with Pb9 epitope (right panels). (**B**) Memory phenotype of antigen-specific CD8^+^ T cells; CD62L (Y-axis) CD127 (X-axis).
(PDF)

Figure S2 **Sample FACS plots** **Figure 3** **.** Mice were primed by the ID route (top panels) or using microneedle Array F (middle panels) or Array G (lower panels) and boosted by the ID route. (**A**) Magnitude of the multi-functional TNF-α (Y-axis), IFN-γ (X-axis) response in gated live CD8^+^ T cells that were unstimulated (left panels) or stimulated with Pb9 epitope (right panels). (**B**) Memory phenotype of antigen-specific CD8^+^ T cells; CD62L (Y-axis) CD127 (X-axis).
(PDF)

Figure S3 **Total number of antigen-specific CD8^+^ T cells after a prime and prime-boost immunization.** BALB/c mice were immunized with MVA-PbCSP by the intradermal (id) route or using an ImmuPatch device of increasing pore volume and were examined after priming or after and ID boost. The total number of antigen-specific CD8^+^ T cells in spleens were quantified after intracellular cytokine staining (ICS) of IFN-γ, TNF-α, and IL-2 subsequent to stimulation with the immunodominant Pb9 peptide. Data are expressed as the total number of cytokine-secreting CD8^+^ T cells, +/− standard error of the mean (+/−SEM), 2 weeks after a single (**A**) or prime-boost (**B**) immunization. * p<0.05, ** p<0.01, ***p<0.001 compared with ID vaccinated mice in by one way ANOVA.
(PDF)

Figure S4 **Total cell counts in draining lymph nodes post-immunization.** BALB/c mice were immunized with MVA-PbCSP by the ID route or using microneedle array F (‘Patch’). Naive mice were untreated. Mean with individual cell counts in homogenised lymph nodes that were harvested from all mice at 24 hours (top panel) or 48 hours (bottom panel) after immunization was determined using a Coulter counter. * p<0.05, ** p<0.01 compared with ID vaccinated mice in by one way ANOVA.
(PDF)

Figure S5 **Distribution of microneedle-delivered fluorescent nanospheres in mouse ear.** A 5 µl solution of red fluorescent nanospheres, 100nm in diameter (Invitrogen) were administered to the ears of anaesthetised BALB/c mice using microneedle array F. Thirty minutes post-administration, animals were culled and ears were removed, preserved and cryosectioned into 10 µm sections. Cell nuclei were stained using DAPI. Samples were examined by fluorescent microscopy (10×).
(PDF)

Figure S6 **Recombinant MVA infects different skin layers when administered by ID or ImmuPatch.** Transgene expression (red fluorescent protein RFP) detected in *ex vivo* pig skin cultures when MVA-RFP (1×10^6^pfu) is delivered by ID or microneedle array G. Freshly excised pig skin was setup in a short-term *ex vivo* culture. MVA-RFP was injected intradermally or administered using microneedle array G and skin was cultured for 14 hours at 37°C to permit virus infection and transgene expression. Skin was then snap frozen and cryo-sectioned into 10 µm sections. Samples were examined by light microscopy (40×). The site of MVA administration is indicated by arrows. Similar results were obtained in four independent experiments.
(PDF)

Figure S7 **Delivery efficiency of nanospheres into murine skin.** Red fluorescent nanospheres were administered to mice in the same manner as described in Figure S4, using arrays F, C or H or using a flat silicon patch with no microneedles (‘flat’). After 30 minutes, animals were sacrificed and the outside of the ears were swabbed with wet cotton wool to remove beads that were on the skin surface. Ears were homogenised in a HCl/Tween80/PBS solution (1.0∶0.1∶0.07 v/v/v). The homogenate was centrifuged at 1400rpm for 3 minutes and the fluorescence in the supernatant was determined and compared to the fluorescence present in the original nanosphere solution administered to mice. Mean (+/− SEM) with individual percentage fluorescence recovered from inside the ear is represented for each group. Mean (+/− SEM) for Flat patch = 0.96 (0.14)%; Patch F = 3.17 (0.46)%; Patch C = 2.58 (0.60)%; Patch H = 2.87 (0.30)%.
(PDF)
