# Supplementary figures and images for: Microneedle Array Design Determines the Induction of Protective Memory CD8+ T Cell Responses Induced by a Recombinant Live Malaria Vaccine in Mice
Source: PLoS One. 2011 Jul 25;6(7):e22442. doi: 10.1371/journal.pone.0022442 (PMC3143140; doi:10.1371/journal.pone.0022442)

Supporting Figure S1A

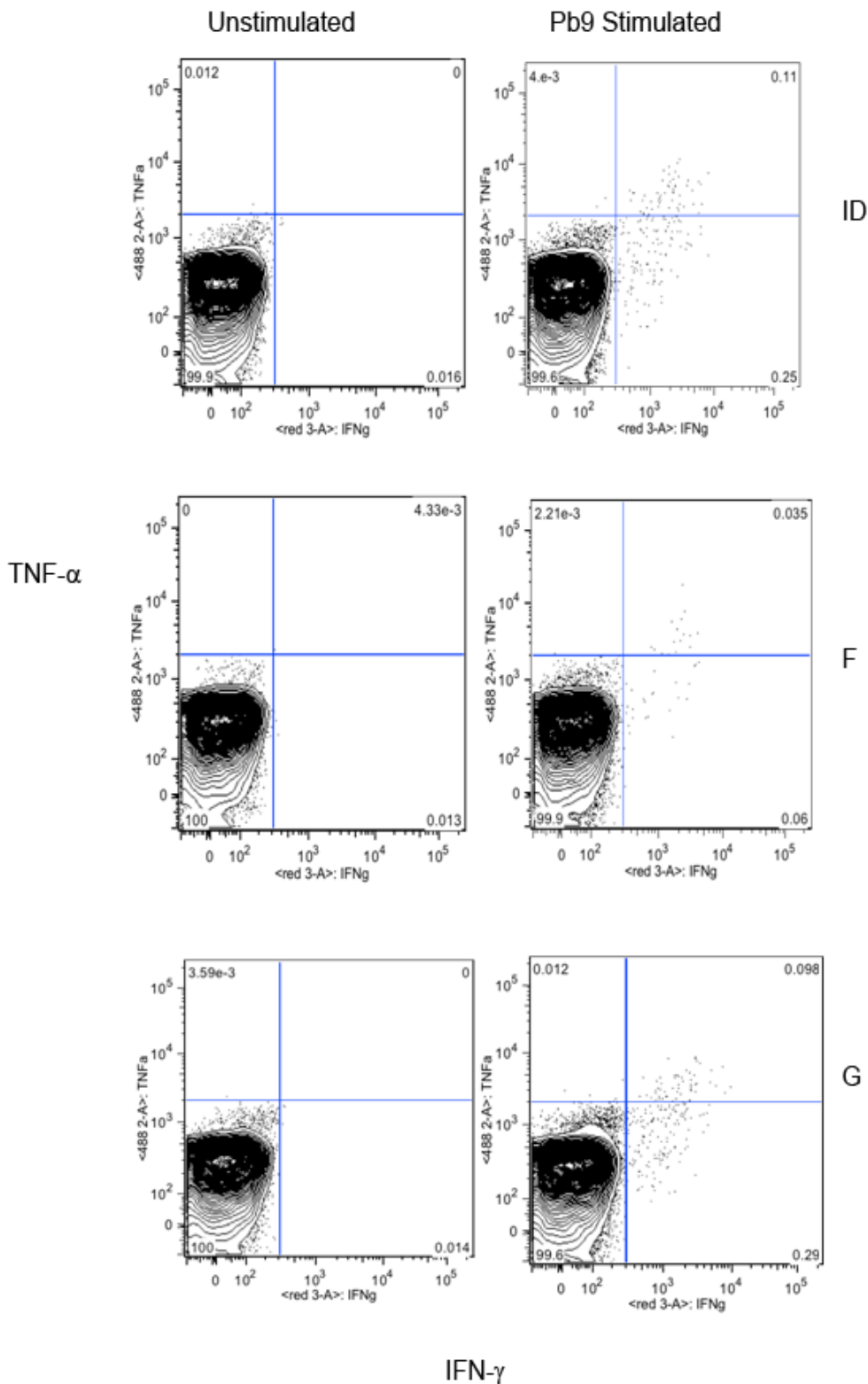

Supporting Figure S1B

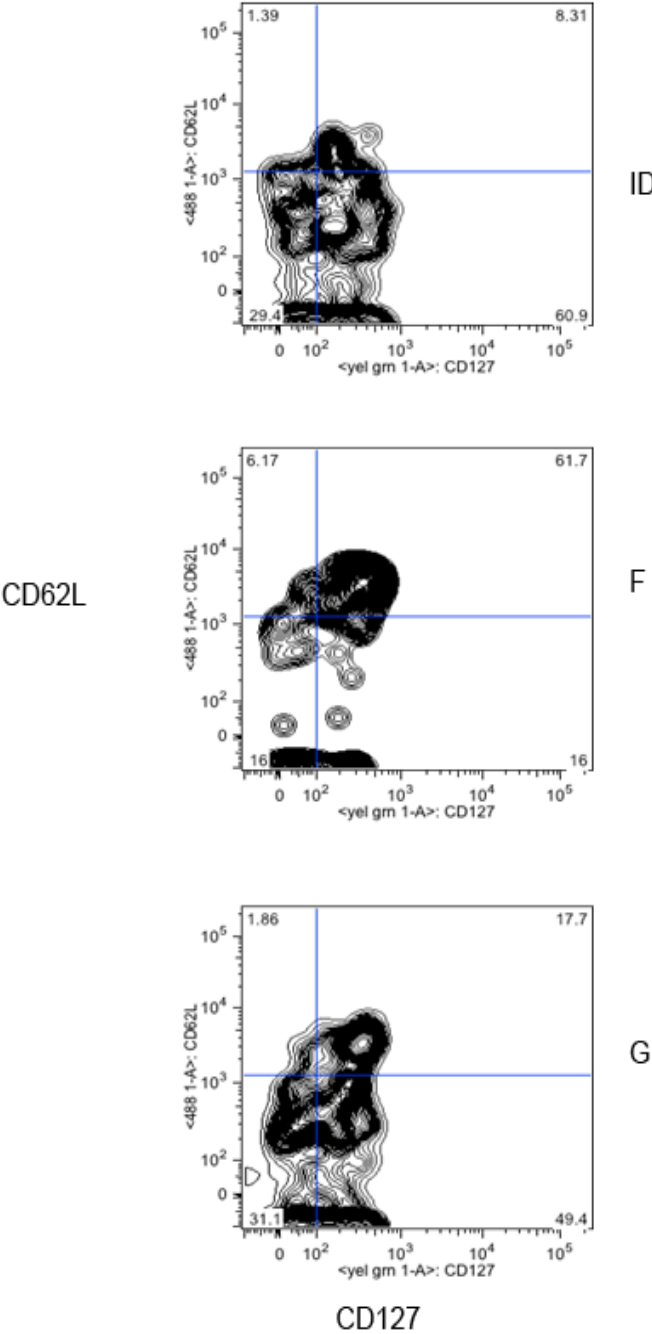

Supplement: Figure S1 — Sample FACS plots Figure 2 . Mice were immunized by the ID route (top panels) or using microneedle Array F (middle panels) or Array G (lower panels). (A) Magnitude of the multi-functional TNF-α (Y-axis), IFN-γ (X-axis) response in gated live CD8+ T cells that were unstimulated (left panels) or stimulated with Pb9 epitope (right panels). (B) Memory phenotype of antigen-specific CD8+ T cells; CD62L (Y-axis) CD127 (X-axis). (PDF) [file pone.0022442.s001.pdf]

Supporting Figure S2A

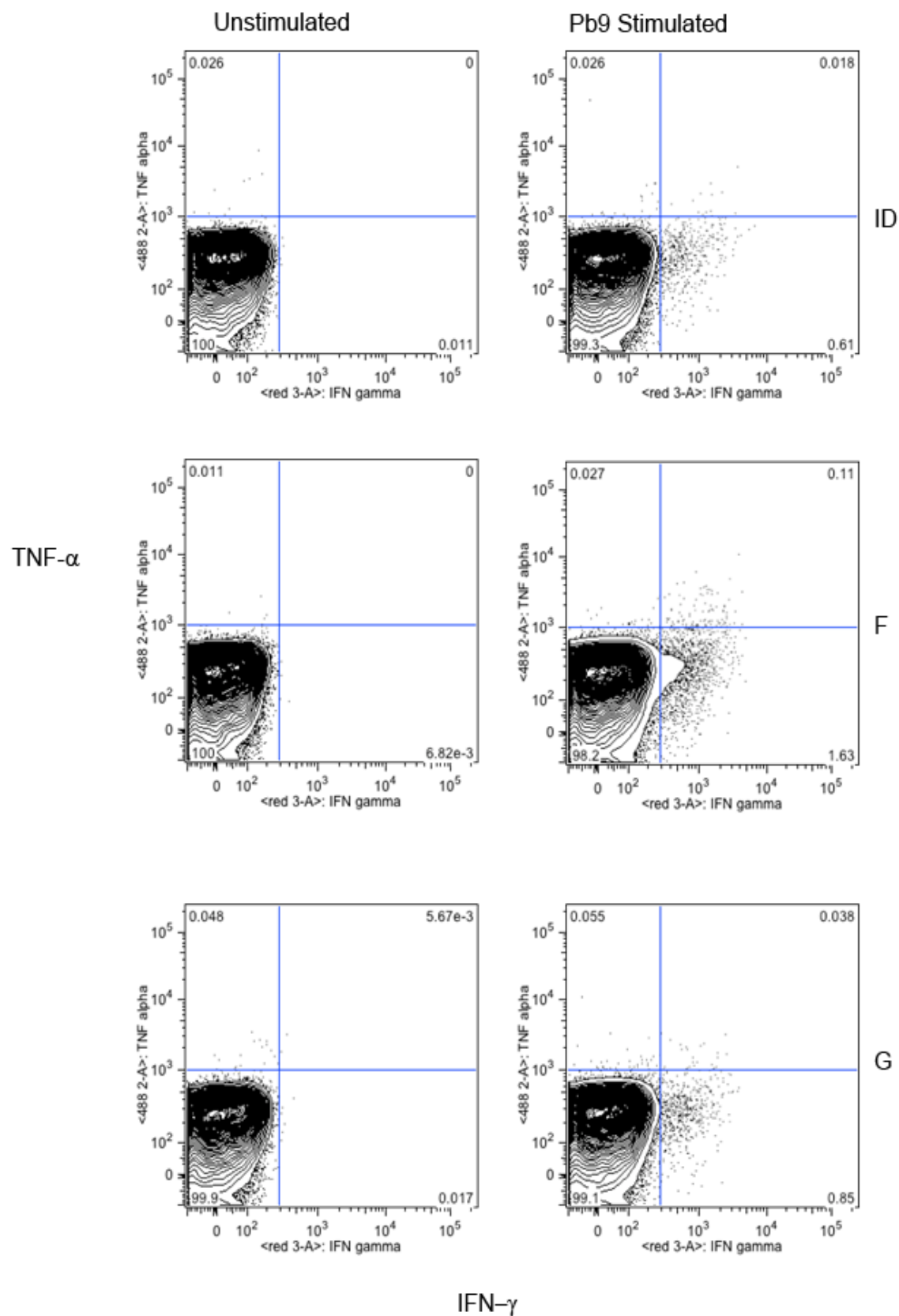

## Supporting Figure S2B

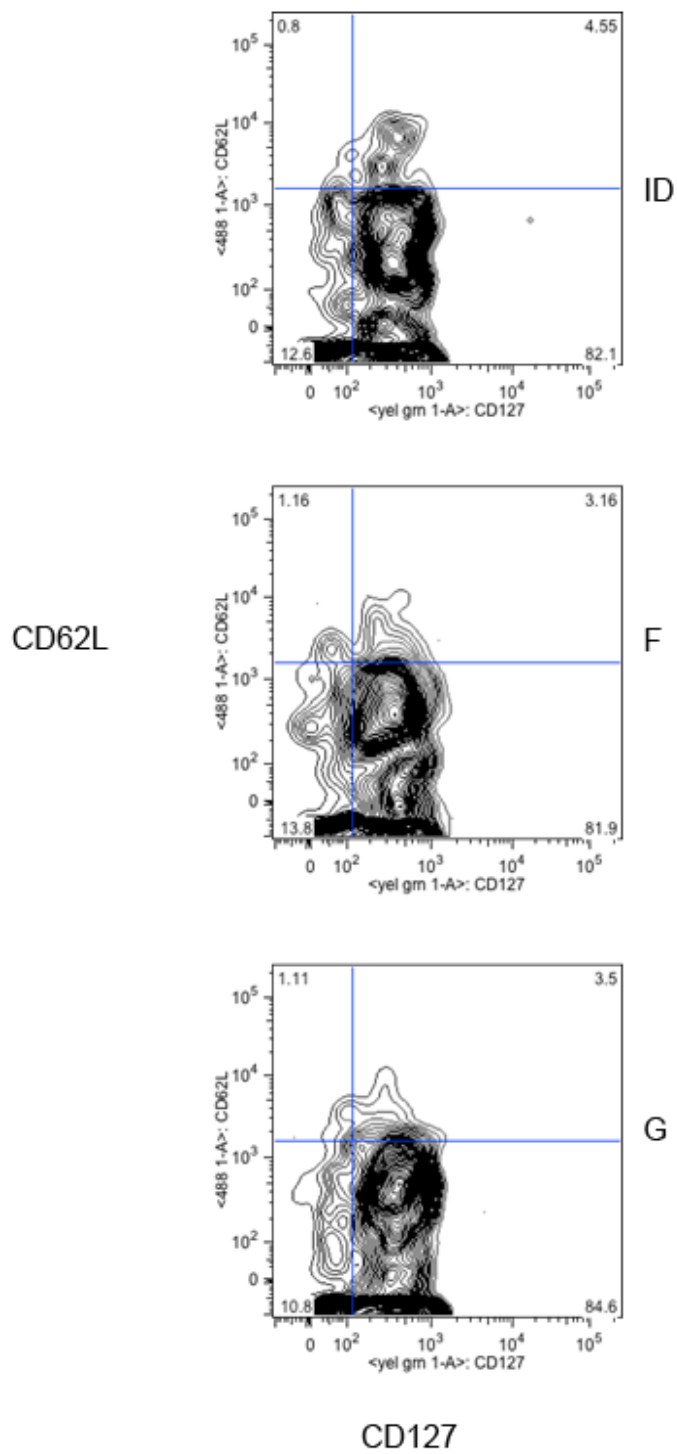

Supplement: Figure S2 — Sample FACS plots Figure 3 . Mice were primed by the ID route (top panels) or using microneedle Array F (middle panels) or Array G (lower panels) and boosted by the ID route. (A) Magnitude of the multi-functional TNF-α (Y-axis), IFN-γ (X-axis) response in gated live CD8+ T cells that were unstimulated (left panels) or stimulated with Pb9 epitope (right panels). (B) Memory phenotype of antigen-specific CD8+ T cells; CD62L (Y-axis) CD127 (X-axis). (PDF) [file pone.0022442.s002.pdf]

Supporting Figure S3

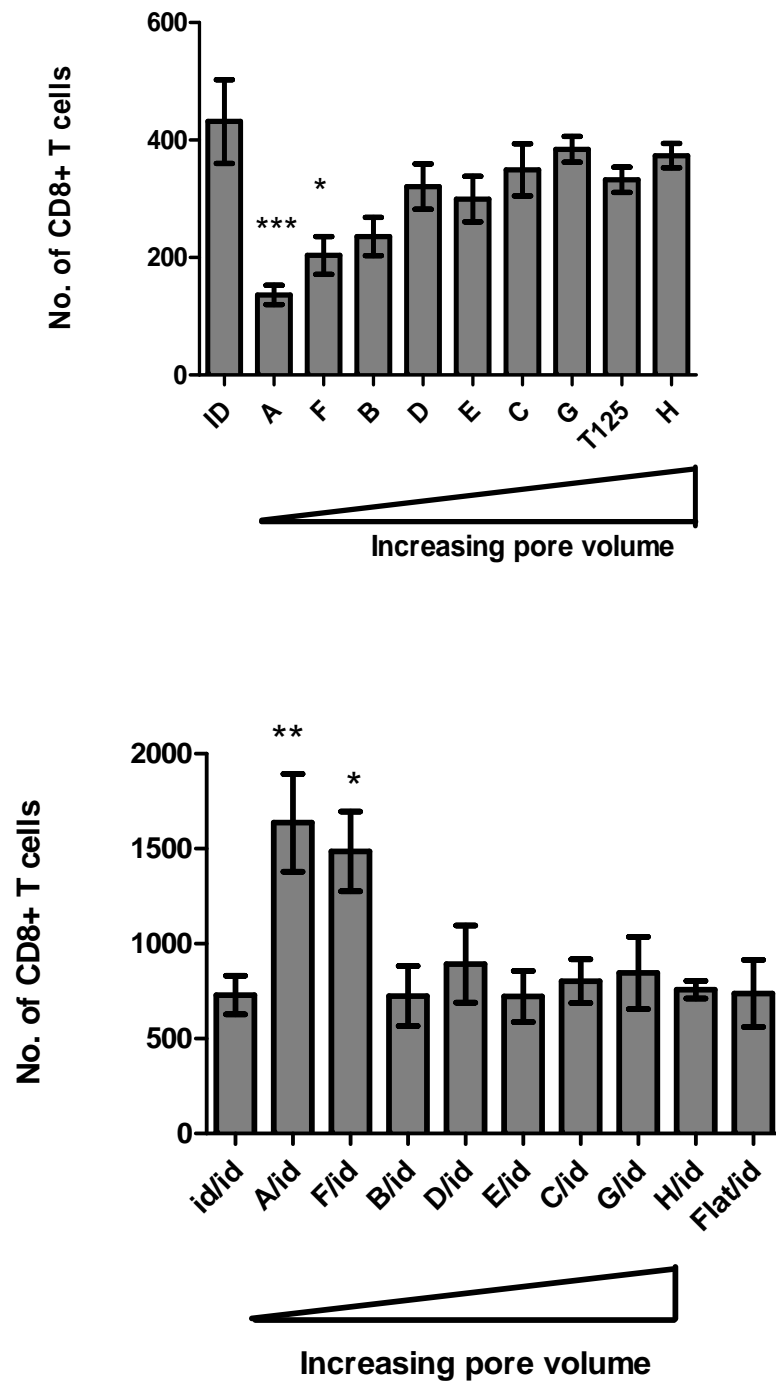

Supplement: Figure S3 — Total number of antigen-specific CD8+ T cells after a prime and prime-boost immunization. BALB/c mice were immunized with MVA-PbCSP by the intradermal (id) route or using an ImmuPatch device of increasing pore volume and were examined after priming or after and ID boost. The total number of antigen-specific CD8+ T cells in spleens were quantified after intracellular cytokine staining (ICS) of IFN-γ, TNF-α, and IL-2 subsequent to stimulation with the immunodominant Pb9 peptide. Data are expressed as the total number of cytokine-secreting CD8+ T cells, +/− standard error of the mean (+/−SEM), 2 weeks after a single (A) or prime-boost (B) immunization. * p<0.05, ** p<0.01, ***p<0.001 compared with ID vaccinated mice in by one way ANOVA. (PDF) [file pone.0022442.s003.pdf]

Supporting Figure S4

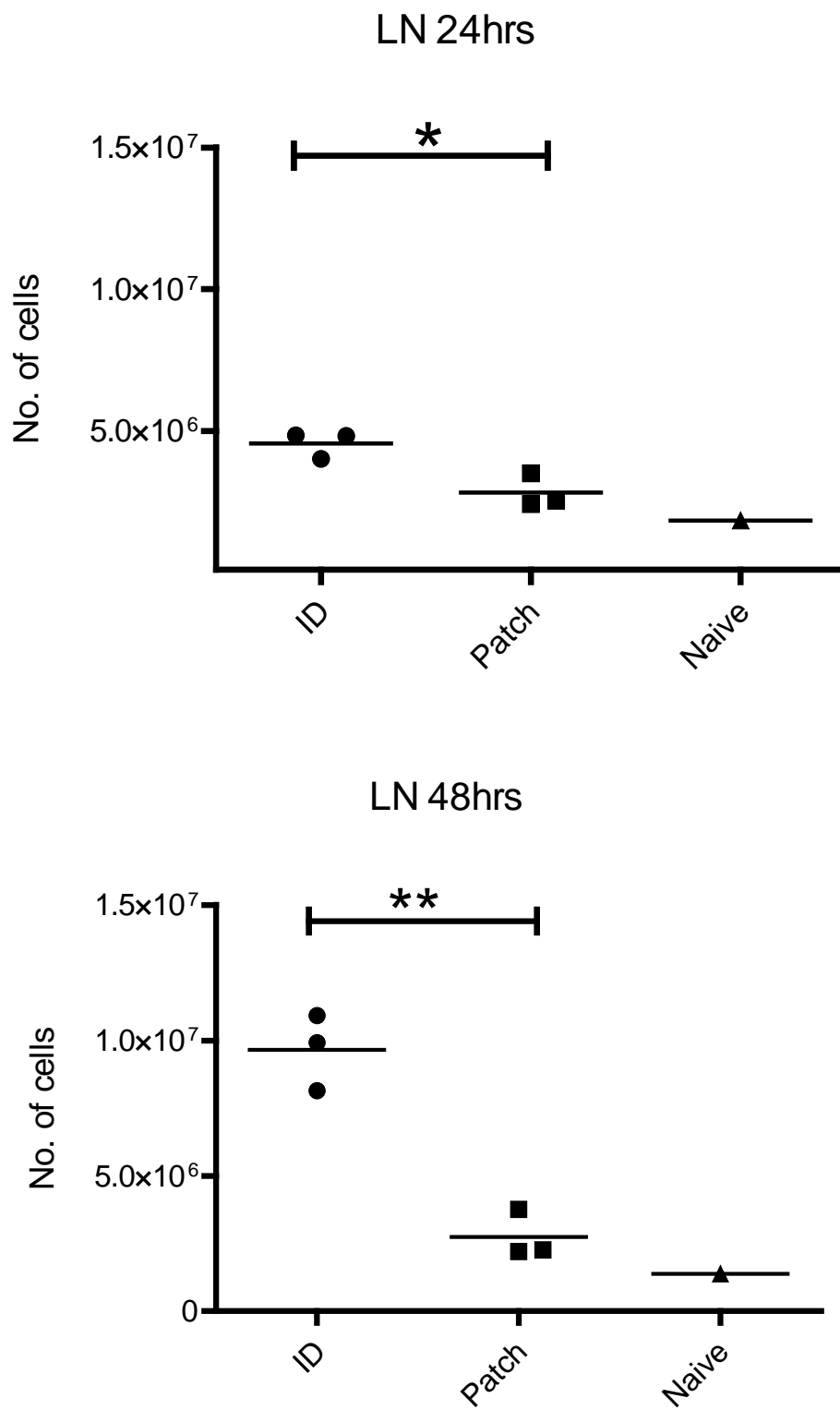

Supplement: Figure S4 — Total cell counts in draining lymph nodes post-immunization. BALB/c mice were immunized with MVA-PbCSP by the ID route or using microneedle array F (‘Patch’). Naive mice were untreated. Mean with individual cell counts in homogenised lymph nodes that were harvested from all mice at 24 hours (top panel) or 48 hours (bottom panel) after immunization was determined using a Coulter counter. * p<0.05, ** p<0.01 compared with ID vaccinated mice in by one way ANOVA. (PDF) [file pone.0022442.s004.pdf]

**Supporting Figure S5**

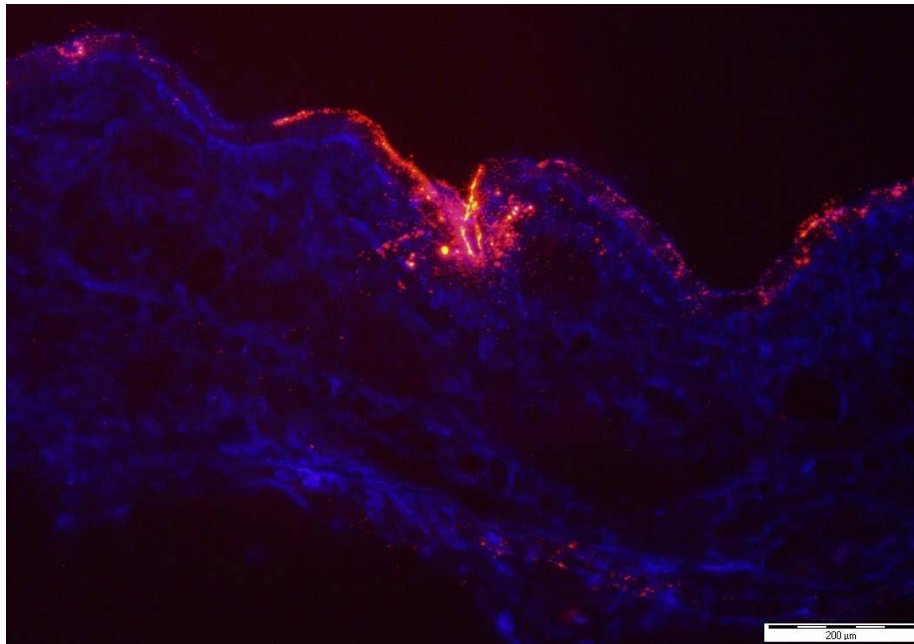

Supplement: Figure S5 — Distribution of microneedle-delivered fluorescent nanospheres in mouse ear. A 5 µl solution of red fluorescent nanospheres, 100nm in diameter (Invitrogen) were administered to the ears of anaesthetised BALB/c mice using microneedle array F. Thirty minutes post-administration, animals were culled and ears were removed, preserved and cryosectioned into 10 µm sections. Cell nuclei were stained using DAPI. Samples were examined by fluorescent microscopy (10×). (PDF) [file pone.0022442.s005.pdf]

**Supporting Figure S6**

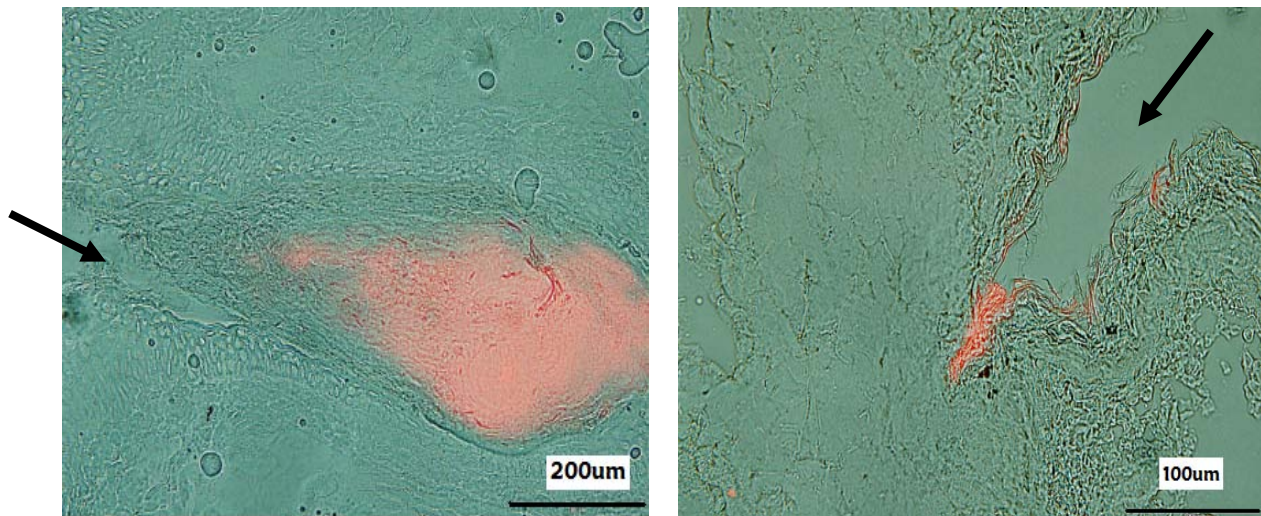

Supplement: Figure S6 — Recombinant MVA infects different skin layers when administered by ID or ImmuPatch. Transgene expression (red fluorescent protein RFP) detected in ex vivo pig skin cultures when MVA-RFP (1×10^6^pfu) is delivered by ID or microneedle array G. Freshly excised pig skin was setup in a short-term ex vivo culture. MVA-RFP was injected intradermally or administered using microneedle array G and skin was cultured for 14 hours at 37°C to permit virus infection and transgene expression. Skin was then snap frozen and cryo-sectioned into 10 µm sections. Samples were examined by light microscopy (40×). The site of MVA administration is indicated by arrows. Similar results were obtained in four independent experiments. (PDF) [file pone.0022442.s006.pdf]

Supporting Figure S7

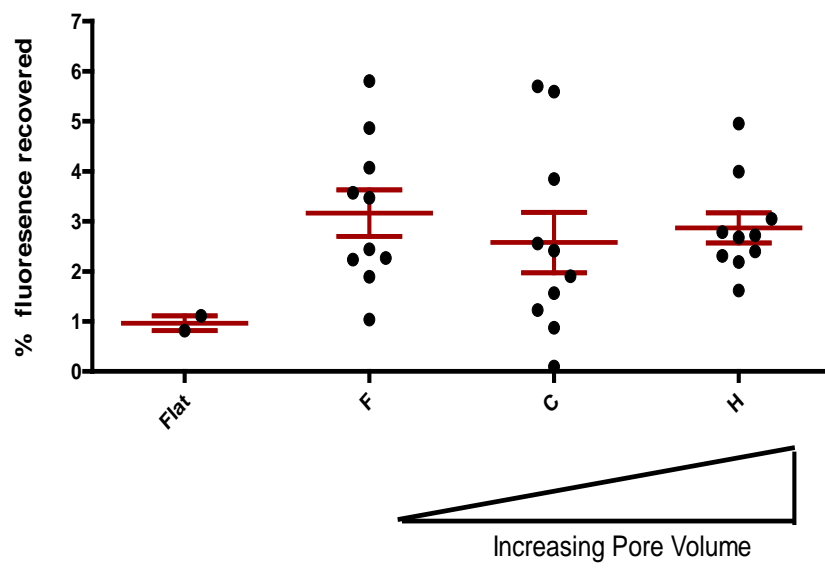

Supplement: Figure S7 — Delivery efficiency of nanospheres into murine skin. Red fluorescent nanospheres were administered to mice in the same manner as described in Figure S4, using arrays F, C or H or using a flat silicon patch with no microneedles (‘flat’). After 30 minutes, animals were sacrificed and the outside of the ears were swabbed with wet cotton wool to remove beads that were on the skin surface. Ears were homogenised in a HCl/Tween80/PBS solution (1.0∶0.1∶0.07 v/v/v). The homogenate was centrifuged at 1400rpm for 3 minutes and the fluorescence in the supernatant was determined and compared to the fluorescence present in the original nanosphere solution administered to mice. Mean (+/− SEM) with individual percentage fluorescence recovered from inside the ear is represented for each group. Mean (+/− SEM) for Flat patch = 0.96 (0.14)%; Patch F = 3.17 (0.46)%; Patch C = 2.58 (0.60)%; Patch H = 2.87 (0.30)%. (PDF) [file pone.0022442.s007.pdf]
